# Tracking the impact of translational research in psychiatry: state of the art and perspectives

**DOI:** 10.1186/1479-5876-10-175

**Published:** 2012-08-28

**Authors:** Rodrigo Machado-Vieira

**Affiliations:** 1 Institute and Department of Psychiatry, Laboratory of Neuroscience, LIM27, University of Sao Paulo, Rua Ovidio Pires de Campos, 785, Sao Paulo, SP, Brazil

**Keywords:** Translational, Psychiatry, Personalized, Treatment, Human, Animal, Research, Biomarker

## Abstract

Personalized treatments have become a primary goal in translational psychiatric research. They include the identification of neural circuits associated with psychiatric disorders and definition of treatment according to individual characteristics. Many new tools and technologies have been developed but further efforts are required to provide clues on how these scientific advances in psychiatry may be translated into more effective therapeutic approaches. Obstacles to the progress of translational psychiatry also involve numerous scientific, financial, ethical, logistics and regulatory aspects. Also, the goal of DSM-5 to expand “signs and symptoms” classification to incorporate biological measures may help the development of new multifactorial and dimensional models able to better understand the pathophysiology of psychiatric disorders and develop improved treatments. Finally, a better understanding on the significant response variability, cognitive functioning, role of comorbidities and treatment-resistant cases are critical for the development of prevention and intervention strategies that are more effective.

## Introduction

Translational research is at the forefront of contemporary psychiatric research. The term “translational” was first cited in the PubMed in 1993, characterizing the gene *BRCA1* and its immediate applications in early detection and treatment of breast cancer [1]. This definition was cited few times throughout the 1990s until 2000, when the term started to be quoted in hundreds of articles every year. Although the term "translational" has various definitions, all focus on a better understanding of the pathophysiology and development of new diagnostic tests, aiming to develop better treatments for specific diseases.

The term translational can be also characterized as the process of obtaining benefit for patients by converting scientific discoveries from preclinical research into clinical applications, with the goal of improving health parameters, consequently decreasing morbidity and mortality [2]. Translational research also refers to those activities conducted to bridge the gap between drug discovery in preclinical models and drug development in humans. It has been used to refer to the entire enterprise of medicine: “from bench to bedside and from bedside to bench” [3,4].

Historically, in the 1950s and '60s, basic and clinical researches were largely interconnected in research institutions such as the National Institutes of Health (NIH), USA and academic institutions worldwide. Biomedical research was performed mostly in laboratories by medical scientists, who also treated patients. In the 1970s, this model was adapted due to the rapid development of molecular biology techniques. In this period, clinical and basic research began to be put apart as two distinct areas of research, also having different staff members. Since that period, most of the biomedical preclinical research (currently considered translational) has been developed by highly skilled PhDs scientists, while physicians lacking or with limited preclinical background have been only conducting clinical studies. Thus, a translational gap has arisen, which means that clinical neuroscience research, previously conducted by physician-scientists, became scarce and limited the communication between basic and clinical scientists.

Besides, other gaps have been identified in translational research. For example, in 2006, a study by Cooksey [5] was hired to conduct an independent assessment on the public funding for health research in the United Kingdom (UK). The report identified two major issues. First, the difficulty to transform basic and clinical research into integrated ideas, concepts and products. An additional gap described refers to the difficulty in translating these ideas, concepts and products into better outcomes and improved treatments for clinical practice. In the same year, the NIH introduced the "Bench to Bedside" award, aiming to encourage collaborations between physicians and basic scientists at different NIH intramural institutes. Other similar public and private initiatives on a larger scale have been developed in recent years, such as the "FDA Critical Path" and the "NIMH Strategic Plan" [6]. The NIH Road Map, with a total budget estimated at 10 billion dollars, was created to provide scientists with the technologies and human resources to enable the more efficient translation, also supporting institutional infrastructure for research centers and departments focusing on translational medicine. Similar initiatives have been developed in diverse countries. These projects aim to introduce innovative tools in pharmacology, machineries and clinical methods able to improve our understanding on the pathophysiological mechanisms and to develop new and better treatments. They also objective to increase efficacy rates of clinical trials phase 2 [2], which involves continuous investigation of potential therapeutic targets and biomarkers in humans, as well as the evaluation of therapeutic index and cost-effectiveness parameters.

Translational research in psychiatry directly involves the concept of personalized medicine, which aims to identify the more accurate individual treatment, based on clinical, genetic, genomic and environmental information. In the last decade, progresses in this area have directly involved the validation of new biomarkers with potential clinical relevance. This area has provided a wide range of scientific progresses using tools and technologies, which integrate basic research information with clinical practice. These biomarkers target on the measurement of physiological and pathological processes, as well as focus on the prediction of pharmacological response to a particular treatment [7].

Biomarkers are cornerstones in the discovery and development of new treatments, ranging from preclinical studies to clinical trials. In 1998, the NIH Biomarkers Definitions Working Group defined a biomarker as “a characteristic that is objectively measured and evaluated as an indicator of normal biological processes, pathogenic processes, or pharmacologic responses to a therapeutic intervention [8]. Another important definition is surrogate biomarker, characterized as “a laboratory or physical sign that is used in therapeutic trials as a substitute for a clinically meaningful endpoint, that is a measure of a how a patient feels, functions, or survives and that is expected to predict the effect of therapy”. Whereas surrogate biomarkers substitute for clinical endpoints, other biomarkers are only adjunct to clinical endpoints [3,4].

**Figure 1:**
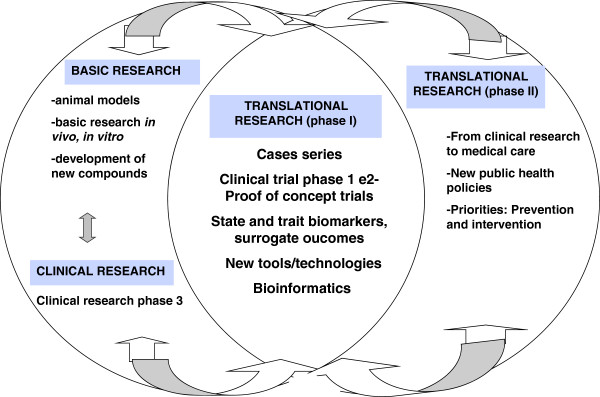
**Interface between basic and clinical research in mood disorders translational research, divided in phase I (translacional research *per se* ) e phase II (translating research advances into population benefits and improved health system).** Translational research in psychiatry involves the development of useful animal behavioral models of psychiatric disorders and preclinical *in vitro/vivo* studies using brain cells. These are the first steps to test new compounds, which evaluate potential mechanisms of action and behavioral effects, focusing on the evaluation of predictive validity. When validated, these new agents are tested in phase 1 translational studies (pharmacokinetics, dose and tolerability), which together with clinical trial phase 2 studies (proof of concept trial evaluating efficacy). These two step use new tools and other technological advances (e.g. bioinformatics), also searching for potential biomarkers (preferentially in early phases). The next step in the translational paradigm involves the approval in phase 3 and posterior application of new public health policies focusing on prevention and early intervention in mental disorders.

Biomarkers aim to provide evidences supporting the concept that certain targets have therapeutic relevance in psychiatric disorders. Discovery, validation and application of biomarkers at all stages of drug development involve different steps. In the search of a new effective compound and identification of its targets (e.g. biomarkers), preclinical studies are followed by clinical safety studies (Phase 1) and efficacy and tolerability (Phase 2 and 3) (Figure 1). Subsequently, Phase 4 involves post-approval studies after marketing the drug to explore other indications, also evaluating effectiveness and safety in daily clinical practice. However, Phase 4 studies do not provide relevant answers in translational research and drug development in psychiatric research.

The pursuit of scientifically based therapeutic decision can be considered a key objective in translational research in psychiatry. Nevertheless, despite these recent advances, only few agents commercially available have arisen from these translational strategies in psychiatry in the last decade, associated with diverse challenges and barriers. For instance, lack of funding, high costs, inadequate samples and conflicts of interest are common limitations. Furthermore, fragmented infrastructure, shortage of qualified researchers, incompatible databases and lack of technical support are important barriers to be overcome in order to increase effectiveness in translational psychiatric research.

At the same time, new methods and disciplines in translational psychiatry have increased the number of new potential therapeutic strategies in the continuum bench to bedside. Recent advances in genomics, proteomics, metabolomics and others have provided advances ad promising perspectives in this area. The model proposed by the National Institute of Mental Health (NIMH) reinforces the need to identify neural circuits associated with psychiatric disorders, as well as to detect early manifestations associated with increased risk for illness, even before the onset of cognitive and behavioral changes. This model also aims to customize services based on individual responses, as well as foster the optimized use of effective psychosocial interventions [6]. Other examples of initiatives in translational psychiatry include the National Institutes for Health Research (NIHR) and industry cooperation to create standardized batteries for assessment of effects of treatments on cognition, such as the MATRICS initiative in schizophrenia [9].

There is a recent trend in translational psychiatry to define at risk populations studying surrogate populations (e.g. relatives of patients or patients with mild forms of the disorder). This approach provides the opportunity for small proof-of-concept Phase 1 trials of novel medications [4,10,11]. For instance, the evaluation of surrogate populations in clinical trials includes subjects with schizotypal personality instead of schizophrenia [9]. Another development is the use of pharmacological agents to induce states that mimic some of the disease features in healthy volunteers (e.g. drug-induced psychoses with amphetamine) [12]. Schizotypal personality presents cognitive deficits, positive and negative symptoms and anhedonia, also considered a risk factor for psychosis [13]. Another example includes neurokinin NK1 antagonists, which showed promising trends in preclinical and initial clinical studies, but not subsequently replicated in Phase 3 trials [14,15].

However, the implementation of this approach still requires adaptation of the currently used phase 1 scheme, and could have a significant impact on the success rates of clinical research in psychiatry. Overall, these approaches may help to limit erroneous therapeutic indication and/or in incorrect doses by testing the likelihood that a drug would be effective in patients and represent a valuable model of psychiatric disorders in Phase 1 trials [12,16].

### Challenges in translational research in psychiatry

The development of a new treatment may take up to 20 years. Obstacles to the progress of psychiatry also involve numerous scientific, financial, ethical, logistics and regulatory issues. Translational approaches in other areas of medicine seem much more developed than psychiatry. In oncology, for example, available biomarkers have been clearly associated with course, prognosis and treatment response. Furthermore, in many cases, they strongly guide the therapeutic decision. Meanwhile, psychiatric disorders present heterogeneous symptomatology, cognitive functioning and comorbidities, also involving a wide range of genetic and environmental risk factors. Thus, translation has a high level of complexity and encompasses a very long path from basic neurosciences to patient care.

In the last decades, preclinical research in psychiatry has arisen due to the obvious inaccessibility of human brain *in vivo* for biochemical and molecular studies. It is important to mention that basic research, especially animal models, may help to limit the number of potential drug candidates that may fail in human trials. Animal models in psychiatric research use a neurobiological tool or a disease model, involving an ample range of behavioral assays [17]. However animal models still have several limitations *per se*. For instance, it is challenging to translate higher cognitive functions and complex affective states (e.g. the switch process in bipolar disorder) [18] that differentiate humans from animals [19]. Besides, with the advent of recent techniques in human studies including neuroimaging *in vivo* studies (e.g. positron tomography-PET, proton spectroscopy-MRS), animal models have been gradually competing with new techniques able to directly evaluate human samples.

Noteworthy, recent advances on techniques and tools for human translational studies come from the field of genetics, neuroimaging, psychopharmacology, neurophysiology, neuropathology, with special relevance for the fronto-striatal-limbic structures and their functions. Despite current development, other potential therapeutic targets than monoamines in translational psychiatric research are still scarce; studies on potential new drugs that would exert their therapeutic effects by targeting identified pathways and circuits using cellular and molecular biology studies require further examination, including the use of human genome sequencing and high throughput technologies. New targets beyond monoamines have been associated with rapid onset of therapeutic actions and improvement in treatment-resistant cases [20]. These new targets include CRF and glucocorticoids receptor antagonists, vasopressin receptor antagonists, glutamate modulators, opioid receptor modulators, histone deacetylase inhibitors, among others [21,22]. Potential reasons limiting the progression of new treatments from basic research to clinical include significant behavioral of a compound effect only in animals but not in humans, treatment given to the wrong subject (diagnosis) and inadequate dose [3,4]. It is important to mention that many treatments showing efficacy in certain psychiatric disorders were first clinically used for other indications (e.g. anticonvulsants in bipolar disorder).

In the same context, the study of endophenotypes may be considered a strategic area in translational research and drug development in psychiatry. Endophenotypes involve the identification of “downstream” traits or facets of clinical phenotypes, as well as the “upstream” consequences of genetic vulnerability to specific psychiatric disorders [23]. Endophenotypes characterize more stable phenotypes with a clear genetic connection (not involving symptoms that fluctuate over time).

Drug discovery in psychiatry has recently focused on the mechanisms underlying cellular vulnerability and resilience targeting beyond monoamines. These new translational approaches in psychiatry may stimulate the called 'fishing expedition'. Instead of using the classical hypothesis-driven approach, they focus on hypotheses-generating experiments, which are much broader and sometimes even difficult to interpret. However, at the same time, this new approach might provide the identification of new potential therapeutic targets beyond monoamines. The question whether expanding, using “fishing expedition” aiming to discover unexpected key therapeutic targets or limiting using hypothesis-driven studies remains controversial. Since 80% of human 25,000 genes have some effect on the brain, the use of hypothesis-generating approaches may increase the level of complexity [24]. In this context, recent studies have generated such a volume of potential new targets that the pharmaceutical industry and academic centers are having trouble "digesting" all new data, not being able yet to fully *“*sift the wheat from the chaff."

It has been also emphasized the importance to design clinical trials that estimate chances of success in initial clinical development, proposed as “quick win, quick kill” (Eli Lilly); this concept focus on the early identification of molecules that will potentially fail or those having the greatest potential for success in phase 1 or early phase 2 [25]. Biomarkers also play a critical role in this strategy. In drug development by pharmaceutical industry, the main reason of trial failure is lack of efficacy and safety issues, responsible for approximately 30% and 20% respectively of all failure [26]. Also, a reduced rate of return on investment has led a number of pharmaceutical companies to cut expenses and limit new projects on psychiatry as a therapeutic area. Two major companies, GlaxoSmithKline and AstraZeneca [27], recently reduced significantly basic research aimed at developing new agents for psychiatric disorders.

Pioneering studies identifying loss of neuroprotection (neurons and glial cells) in psychiatric disorders involve dysfunction in multiple neurotransmitter systems, second messengers and gene expression. Postmortem brain studies, animal models and central (CSF) and peripheral biomarkers have provided important knowledge in the context of neuroplasticity. These targets may involve the use of approaches such as cell biology (cell membrane and organelles) and molecular (second messengers and signal transduction) tools. Also, changes in neurotrophic factors, oxidative stress parameters, inflammatory biomarkers and intracellular plasticity cascades in the periphery have been consistently described in psychiatric disorders. However, further studies using live human peripheral cells are still important using more homogeneous samples and drug-naïve subjects. Also, in neuroimaging studies, recent techniques such as diffusion tensor imaging (DTI) allow a comprehensive assessment of functional connectivity and identification of biophysical characteristics of white matter for mapping potential pathways involved in human behavior. Likewise, MRS, PET and functional studies have increased the relevance of human studies. These techniques allow the identification of changes in transporters and receptors uptake, also providing quantification of cerebral blood flow, glucose metabolism and others. Finally, measurements in resting state or during cognitive and affective tasks provide important clues on the pathophysiology of psychiatric disorders.

### Perspectives on the translational psychiatric research

Translational research in psychiatry covers a wide range of scientific areas, tools and technologies that aim to integrate basic research with clinical practice and to develop more effective treatments. Important questions in this context address are if advances in the field have been translated into better therapies, as well as the potential roles of biomarkers in drug development.

At present, further efforts are also required to develop a cadre of highly qualified physician investigators who can effectively lead multidisciplinary translational research groups in psychiatry, integrating genomic science, molecular neurobiology, and clinical investigation. At the same time, there is an urgency to craft researchers with technical skills able to master the intricacy and advances in genomics, proteomics, and metabolomics, as well as the related laboratory validation methods and adequate interpretation of results. Also, standardization and technical validation of biomarkers in psychiatry have been modest, and still require more exploratory studies in early drug development.

The development of translational research centers is critical, which are expected to involve researchers with experience in both basic and clinical sciences. Not being able to find researchers having this profile, translational research in psychiatry should provide an environment that allows a direct communication between basic scientists and clinicians, aiming to test hypotheses and develop new models and methods. In this context, the organization of academic teams in translational research models may provide a continuous dialogue between basic and clinical researchers, with exchange of experience among junior, mid-career and senior researchers and development of centers of excellence in translational psychiatric research.

Collaborations between academia and the private sector may involve significant resources from pharmaceutical and biotechnology companies, aiming to conduct proof of concept clinical studies in academic settings in order to develop new effective treatments. Currently, some pharmaceutical companies have active departments of translational psychiatry, while others did create drug discovery programs in collaboration with universities and small biotech/biopharma start-up companies. Mostly, industry has backed away from psychiatry until new mechanisms, targets and compounds may increase the probability of success. For instance, in 2007, a consortium agreement called P1vital started collaboration between industry (AstraZeneca, GlaxoSmithKline, Lundbeck, Organon and Wyeth) and five academic clinical psychopharmacology groups at the University of Bristol, Cardiff University, the Institute of Psychiatry-London, the University of Manchester and the University of Oxford. This consortium aimed to define valuable healthy volunteer models able to detect the efficacy of novel compounds for phase 1 clinical studies [13].

The main mission is to improve the predictive validity of potential new therapeutic agents, from preclinical studies and throughout different stages of drug development to clinical use and commercialization.

Translational research in psychiatry involves a bidirectional continuum. The physical proximity of preclinical and clinical research centers can facilitate logistics and communication between researchers. Also, results from basic research may indicate new clinical applications, but the reverse should also be considered, ie, problems and issues raised by clinicians may bring relevant answers to better design and hypothesize basic research projects.

Translational psychiatry is also expected to integrate dimensional approaches to the current categorical diagnosis, which is going to be preliminarily shown in the DSM-5. The goal of DSM-5 to expand “signs and symptoms” classification incorporating biological measures may help to develop new multifactorial and dimensional models able to better understand the pathophysiology of psychiatric disorders. These models may provide insights on the significant response rates variability and high refractory rates in psychoses and affective disorders. In other words, DSM-5 may help to develop translational research in psychiatry by using dimensional approaches within and across diagnostic groups in order to decrease clinical heterogeneity and improve diagnostic validity. The integration of dimensions with diagnostic categories represents a promising and potentially transformative approach to DSM-5 it simultaneously addresses DSM-IV's clinical short-comings and craft novel pathways for research in neurobiology and genetics [28].

The need for personalized psychiatry is critical. For instance, pharmacogenomics has shown utility in the pursuit of personalized approaches by the identification of polymorphisms potentially associated with individual clinical response and tolerability [29,30]. Also, new technologies such as microarrays, gene expression profiling using modern platforms may provide concomitant evaluation of a large number of genes with potential clinical relevance with much lower cost than few years ago. Promising disciplines in biomedical informatics in the translational paradigm also include bioinformatics, imaging informatics, clinical informatics and public health information [31]. Likewise, recent advances in biostatistics have provided new tools for dealing with multiple reading problems and complex databases. These approaches may help to define who may benefit from and who may experience side effects of a specific treatment.

Similarly, score systems for quantitative biomarkers have also been proposed [19] and may help to decide about future investments in phase 3 trials. Also, evaluation of longitudinal clinical outcomes is important, including long-term remission. This approach may require a longer period of monitoring, and allow the identification of predictors of response (only assessed at the beginning of a study) and most importantly, surrogate outcomes (e.g. biomarkers also evaluated prospectively during the clinical follow-up and associated with clinical response). Surrogate outcomes represent a more consistent and reliable neurobiological data in order to “validate” constructs. Thus, the evaluation of psychopathology longitudinally associated with targeted biomarkers under a specific treatment may predict who is most likely to respond best, in the context of personalized psychiatry.

Other aspects are relevant. The search for funding in translational research is a major challenge to the academic community. Also, it is important to note that the translational paradigm also considers relevant findings with negative results. Furthermore, definition of homogeneous criteria about the collection, processing and storage and retrieval of biological samples, as well as development of scoring systems for assessment of biomarkers are warranted. Finally, researchers need to focus on the identification of scientific barriers, as well as financial, ethical, regulatory and operational challenges, including intellectual property and patent legislation, also offering creative solutions.

## Conclusions

Measuring the results of translational research in psychiatry does not rely only on the number of publications. The potential success also should take into consideration the number of citations, clinical trials, collaborations, patents, and most importantly, the potential benefit to patients (Figure 1). At the same time, there is a critical need to “post-translate” results from clinical research to medical practice, which still is limited by the long time that takes to transform preclinical studies into medical care.

Overall, the development of new tools and concepts may optimize many aspects of drug discovery. These may help to provide more consistent findings using smaller samples in clinical studies, also decreasing trials duration and cost. Additionally, new approaches in translational research and personalized psychiatry may help to increase efficacy rates, tolerability and expand the number of approved therapeutic options in psychiatric disorders, thus limiting empirical treatment decision for a specific psychiatric condition. The most important challenge in translational research and personalized psychiatry is now to translate fundamental discoveries and insights already obtained into better preventive approaches and improved treatments for those who need it most.

## Competing interest

The author declares no conflict of interest.
